# Causal associations between circulating cytokines and risk of sepsis and related outcomes: a two-sample Mendelian randomization study

**DOI:** 10.3389/fimmu.2024.1336586

**Published:** 2024-03-05

**Authors:** Feng Zhi, Jia-wei Ma, Dan-dan Ji, Jie Bao, Qian-qian Li

**Affiliations:** ^1^Department of Critical Care Medicine, Wuxi No.2 People’s Hospital, Jiangnan University Medical Center, Wuxi, China; ^2^Department of Critical Care Medicine, Aheqi County People’s Hospital, Xinjiang, China

**Keywords:** sepsis, circulating cytokines, Mendelian randomization, macrophage inflammatory protein 1 beta, monocyte chemoattractant protein-1

## Abstract

**Introduction:**

Sepsis represents a critical medical condition that arises due to an imbalanced host reaction to infection. Central to its pathophysiology are cytokines. However, observational investigations that explore the interrelationships between circulating cytokines and susceptibility to sepsis frequently encounter challenges pertaining to confounding variables and reverse causality.

**Methods:**

To elucidate the potential causal impact of cytokines on the risk of sepsis, we conducted two-sample Mendelian randomization (MR) analyses. Genetic instruments tied to circulating cytokine concentrations were sourced from genome-wide association studies encompassing 8,293 Finnish participants. We then evaluated their links with sepsis and related outcomes using summary-level data acquired from the UK Biobank, a vast multicenter cohort study involving over 500,000 European participants. Specifically, our data spanned 11,643 sepsis cases and 474,841 controls, with subsets including specific age groups, 28-day mortality, and ICU-related outcomes.

**Results and Discussion:**

MR insights intimated that reduced genetically-predicted interleukin-10 (IL-10) levels causally correlated with a heightened sepsis risk (odds ratio [OR] 0.68, 95% confidence interval [CI] 0.52-0.90, P=0.006). An inverse relationship emerged between monocyte chemoattractant protein-1 (MCP-1) and sepsis-induced mortality. Conversely, elevated macrophage inflammatory protein 1 beta (MIP1B) concentrations were positively linked with both sepsis incidence and associated mortality. These revelations underscore the causal impact of certain circulating cytokines on sepsis susceptibility and its prognosis, hinting at the therapeutic potential of modulating these cytokine levels. Additional research is essential to corroborate these connections.

## Introduction

Sepsis, a severe medical condition characterized by an exaggerated inflammatory response to infection, can result in potential organ dysfunction and fatality (1). Globally, sepsis remains a leading cause of death, accounting for millions of fatalities each year (2). The pathogenesis of sepsis is complex and involves intricate interactions between pro- and anti-inflammatory cytokines (3). Yet, data from observational investigations highlighting the relationships between circulating cytokine concentrations and sepsis vulnerability are prone to issues of confounding and potential reverse causality (4).

Mendelian randomization (MR) analysis employs genetic variants as instrumental proxies to deduce causal associations between amendable exposures and clinical outcomes (5). This method effectively mitigates confounding, given that genetic variants undergo random assortment at conception. Additionally, concerns of reverse causation are alleviated since genotypes are established prior to the manifestation of disease. Numerous preceding MR investigations have delved into the causal implications of inflammatory biomarkers in predisposition to infectious diseases (6, 7). Nevertheless, comprehensive assessment of whether circulating cytokines levels play a causal role in sepsis predisposition is still lacking. Our overarching objective was to glean novel etiological insights into the cytokine mediators integral to sepsis pathogenesis.

## Research design and methods

### Study design

**Figure 1:**
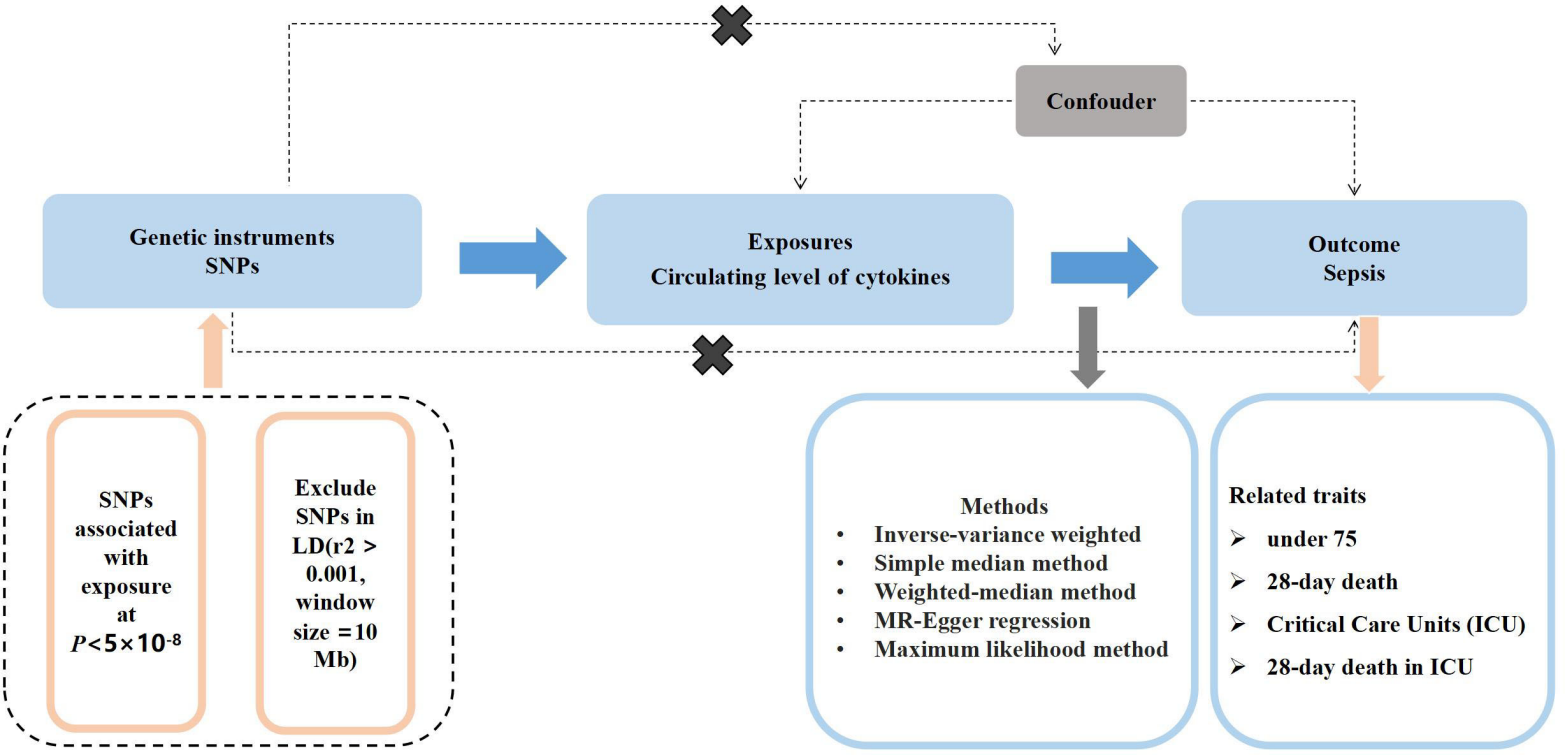
An overall design of the present study.

**Figure 1** shown the overall design of this MR study. Briefly, we selected instrumental variables (IVs) for the circulating level of cytokines from the summary-level data including 8,293 Finnish participants. We then assessed associations of the selected instrumental variables with sepsis in summary-level data of a GWAS meta-analysis of European ancestry. We excluded the single nucleotide polymorphisms (SNPs) associated with two or more phenotypes to assess the robustness of our findings. The characteristics of the summary statistic data for cytokines and sepsis, and further details of SNPs used as instrumental variables were presented in the **Supplementary Tables 1**, **2**.

### Source of outcome

The UK Biobank study, conducted between 2006 and 2010, gathered over 500,000 participants from various centers in the United Kingdom. In this study, sepsis data and its subgroups (including those under 75, 28-day death, Critical Care Units (ICU) cases, and 28-day death in ICU cases) were collected from the IEU Open GWAS using summary-level data sourced from the UK Biobank. The dataset consisted of 11,643, 11,568, 1,896, 1,380, and 347 sepsis cases, along with 474,841, 451,301, 484,588, 429,985, and 431,018 controls, respectively. The analysis of the GWAS data was carried out using Regenie version 2.2.4, with adjustments made for age, sex, chip type, and the first 10 principal components (https://gwas.mrcieu.ac.uk/datasets/ieu-b-4980/).

### Instrumental variables selection

As previously described, for circulating levels of cytokines, summary-level data was drawn from Young Finns Study (YFS) and FINRISK (1997 survey and 2002 survey), comprising a total of 8,293 participants from Finland (8). Participants in the FINRISK study were older (60 years) compared to those in the YFS (36 years). Blood samples for cytokine quantification were collected, with plasma utilized in the FINRISK study and serum in the YFS. A comprehensive analysis of 48 cytokines was conducted in this GWAS study; however, seven cytokines were excluded due to the presence of over 90% missing values. For MR analysis, there are three assumptions that need to be satisfied. First, instrumental variables should be strongly associated with exposure factors. Hence, given the lack of genetic variants reaching genome-wide significance for 13 cytokines, we selected 613 SNPs that reached genome-wide significance (*P* < 5×10^-8^) of the 28 cytokines. In addition, to avoid the influence of linkage disequilibrium and ensure the independence of SNPs, we retained the SNP with the lowest *P*-value at a linkage disequilibrium threshold of r^2^ < 0.01, resulting in total of 183 SNPs (9). Second, IVs must not be statistically associated with confounding factors that affected relationship of exposure-outcome. Finally, genetic variants can only influence outcome occurrence through exposure factors, and we excluded the 16 SNPs associated with one or more cytokines. Finally, we identified 167 SNPs associated with the remaining 28 cytokines, which were subsequently used in the MR analysis. After excluding 19 SNPs missing in the outcome summary-level data, we used the 148 SNPs as instrumental variables in primary MR analyses (**Supplementary Table 3**).

### Statistical analysis

For each cytokine, we determined the proportion of variance explained by the associated primary instrumental variables and assessed the strength of the selected instrumental variables using *F-statistic*s. Following extraction of the effect size and standard error estimates for circulating cytokines and sepsis, we computed the individual MR estimates with the Wald ratio and Delta method. To gauge the connection between genetically determined circulating cytokine levels and sepsis, we aggregated MR estimates across individual SNPs using the inverse-variance weighted (IVW) method. IVW yielded the accurate estimates of causal effects, operating under the assumption that the polytropic effects of genetic variants averaged to zero, and they were independent of the genetic variant-exposure associations (10). The Cochran’s Q test was utilized to measure heterogeneity among the instrumental variables. During the IVW analysis, if significant heterogeneity was detected among the instrumental variables (Cochran’s Q test *P*-value < 0.05), we employed a random-effects model to assess causal associations; otherwise, a fixed-effects model was used. To ensure the robustness of our findings, we conducted sensitivity analyses, including the sample median method, weighted-median method, MR-Egger regression, and maximum likelihood method. Notably, the weighted median estimator provides reliable causal effect estimates when less than 50% of the information is derived from invalid instruments (11). Additionally, the intercept of the MR-Egger regression was employed to assess the presence of directional pleiotropy (a *P*-value for the intercept < 0.05 indicates statistical significance). Besides, the Maximum likelihood method estimates the causal effect by directly maximizing the likelihood, assuming a linear relationship between the exposure and outcome (12).

All MR analyses were done in R (version 3.6.3) using the TwoSampleMR, MendelianRandomization and the MRPRESSO packages.

## Results

**Figure 2:**
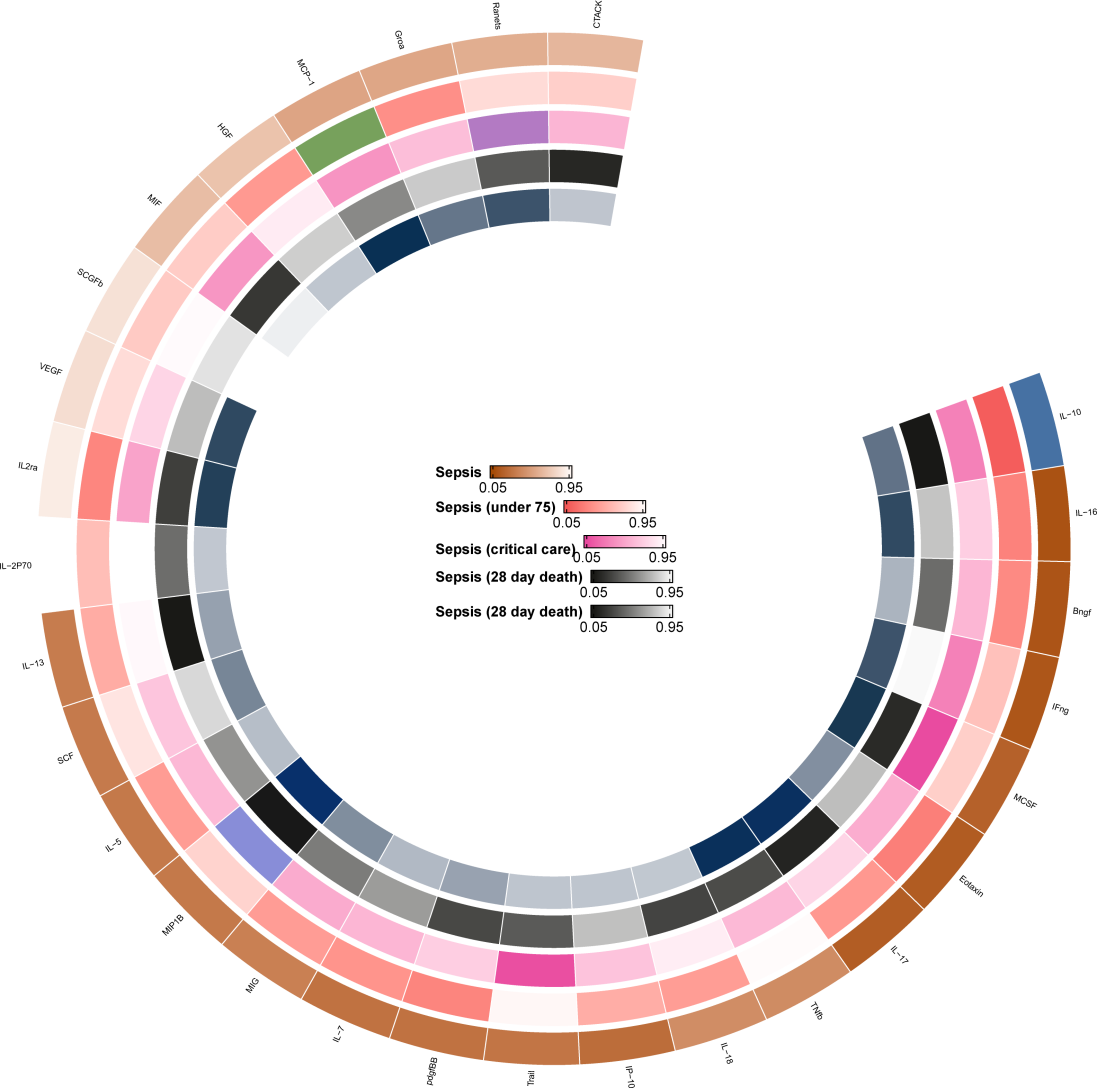
The primary MR analysis between cytokines and sepsis and related outcomes.

**Figure 3:**
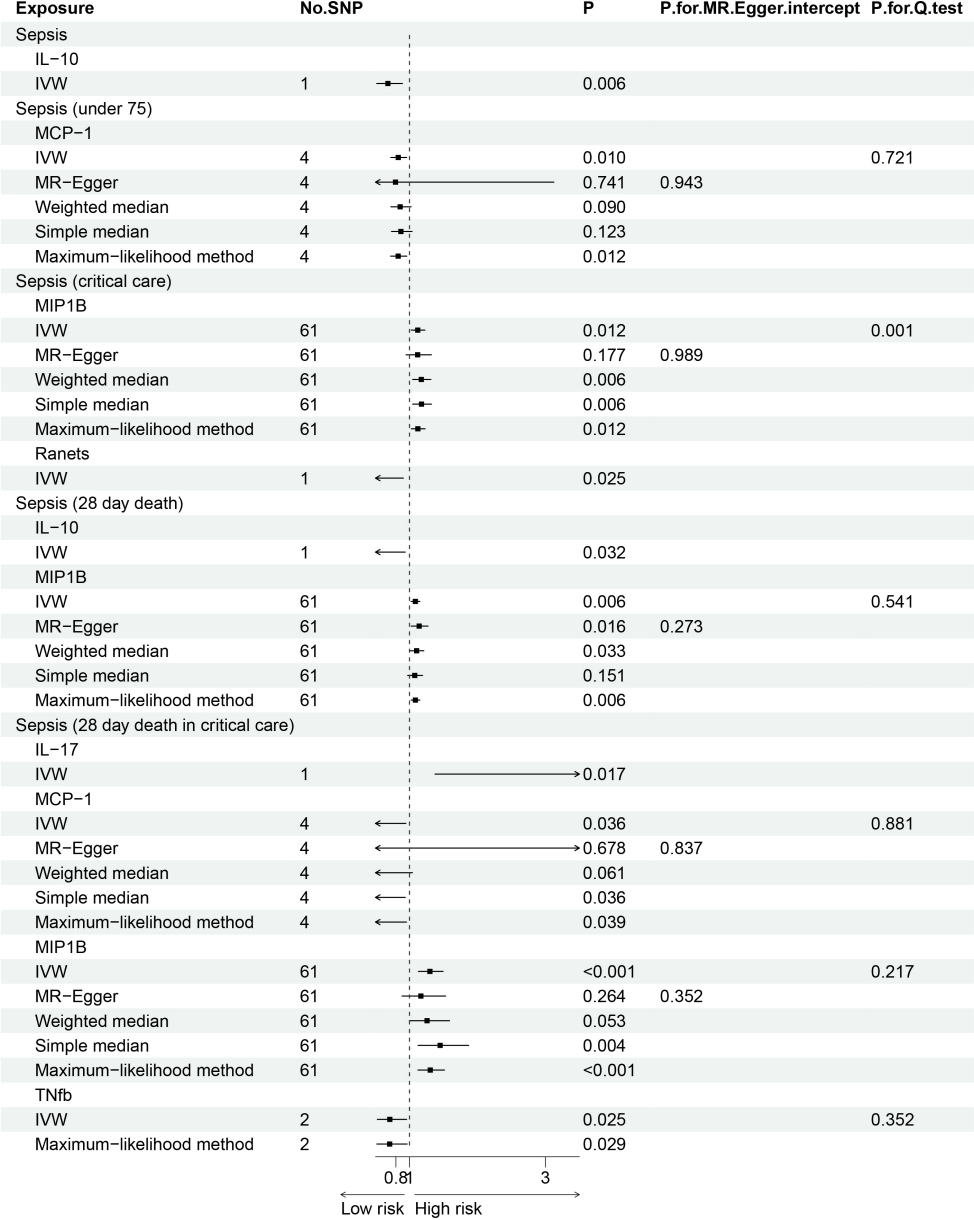
Forest plot of the Mendelian randomization analyses for the associations between circulating cytokines and sepsis and related outcomes.

The *F* statistics for the instrumental variables of 28 cytokines ranged from 28.56 to 789.2 (**Supplementary Table 2**), suggesting that none of them suffer from weak instrument bias. As shown in **Figure 2**, we totally found six cytokines were associated with sepsis and its related traits in the IVW methods. In brief, genetically predicted IL-10 dropped a 32% (OR, 0.68; 95% CI =0.52-0.90, *P* = 0.006) risk of sepsis in the IVW method. We also found a negative effect of MCP-1 level on sepsis (under 75) trait (OR, 0.83; 95% CI =0.73-0.96, *P* = 0.010), and this causality was kept consistent in other approaches, such as Maximum likelihood and Weighted median and simple median (**Figure 3**). The heterogeneity test and pleiotropy test indicated that there no influence for the casual effect. We also found that there was an inverse relationship between MCP-1 and Sepsis (28 day death in critical care) (OR=0.42, 95% CI =0.19-0.95, *P* = 0.036). In addition, MIP1B was found to be associated with a slightly increased risk in Sepsis (28-day death in critical care) (OR=1.30, 95% CI=1.13-1.49, *P*<0.001), Sepsis (critical care) (OR=1.12, 95% CI=1.03-1.23, *P*=0.011) and Sepsis (28-day death) (OR=1.09, 95% CI=1.02-1.15, *P*=0.006). The more results of the association of cytokines with sepsis and its related traits are shown in the **Supplementary Tables 4**-**8**.

In order to confirm the causal association discovered, we performed a reverse MR analysis for the above statistically significant results. We didn’t find any sepsis and its related traits would influence the level of cytokines (**Supplementary Table 9**).

## Discussion

In this two-sample MR study leveraging GWAS summary data of European populations, we found evidence supporting causal effects of genetically predicted circulating IL-10, MCP-1 and MIP1B levels on sepsis risk and related outcomes.

Our results revealed a potentially protective effect of higher IL-10 levels against sepsis. As an important anti-inflammatory cytokine, IL-10 inhibits immune cell function and curbs excessive inflammation (13). The expression of costimulatory molecules by myeloid and lymphoid cells are reduced through IL-10, which reduces the secretion of inflammatory cytokines as well as the production of MHC class II antigens (14). IL-10 further inhibits the synthesis of pro-inflammatory cytokines such as TNF-α, IL-1β, IL-6, IL-8, and IL-12, specifically in macrophages and dendritic cells (15). Diminished production of IL-10 has been correlated with the severity and mortality rates in patients with sepsis (16, 17). Enhancing IL-10 signaling could represent a promising therapeutic approach. Recombinant human IL-10 has shown efficacy in clinical trials for inflammatory conditions like psoriasis, Crohn’s disease, and rheumatoid arthritis (18, 19). However, timing and dosage of IL-10 administration need to be carefully titrated, as sustained immunosuppressive effects can impair pathogen clearance and exacerbate secondary infections.

The protective effects of IL-10 may be mediated through several mechanisms. By limiting antigen presentation and co-stimulatory molecule expression, IL-10 prevents excessive T cell activation and proliferation, reducing tissue damage from uncontrolled inflammation (20). IL-10 also enhances phagocytosis of apoptotic cells and downregulates nitric oxide production, promoting resolution of inflammation (21, 22). Additionally, IL-10 can directly suppress endothelial activation and leukocyte recruitment, attenuating vascular permeability changes that underlie organ dysfunction in sepsis (23, 24).

From a therapeutic perspective, clinical trials evaluating IL-10 supplementation in sepsis patients have yielded inconsistent results. A small phase II trial of recombinant human IL-10 showed improved survival and reversal of shock in subjects with high baseline IL-10 levels (25). However, two subsequent large phase III trials failed to demonstrate mortality benefit with IL-10 administration (26). Several factors may account for the discordant findings. Heterogeneity in sepsis etiology and differences in treatment protocols across studies could influence outcomes. The biphasic temporal response of IL-10 in sepsis should also be considered for optimal timing of IL-10 therapy (27). Early administration may curb hyperinflammation, while later supplementation when immunosuppression predominates can increase secondary infections. IL-10 effects likely depend on the prevailing cytokine milieu, and evaluation of dynamic patient endotypes may help guide precision approaches (28). Overall, the complexities of translating IL-10 modulation into effective sepsis treatments warrant further mechanistic characterization.

We also observed an inverse association of MCP-1 with sepsis mortality, contrasting with the positive effect of MIP1B. MCP-1 (CCL2) and MIP1B (CCL4) both belong to the CC chemokine family and are key chemoattractants regulating leukocyte recruitment and activation (29). However, they trigger divergent downstream pathways that may differentially impact sepsis outcomes. MCP-1 induces a phenotype switch in blood monocytes from an anti-inflammatory IL-10-producing profile to a proinflammatory TNF-α/IL-6-secreting one (30). Its blockade has shown protective effects in animal models of sepsis (31). In contrast, MIP1B preferentially attracts Th1 lymphocytes and stimulates production of interferon-gamma over other proinflammatory cytokines (32, 33). The contrasting effects of MCP-1 and MIP1B on sepsis prognosis warrant further mechanistic characterization.

The mechanisms underlying the opposing effects of MCP-1 and MIP1B on sepsis mortality remain to be fully elucidated. MCP-1 is a major driver of classical monocyte recruitment, which mediate tissue damage in sepsis through inflammatory cytokine production and oxidative stress (34). MIP1B may recruit beneficial Th1 cells that enhance pathogen clearance and resistance to secondary infections (35, 36). MCP-1 also promotes polarization of alternatively activated M2 macrophages, which exhibit impaired bactericidal capacity (37, 38). Differential effects on monocyte and macrophage functional phenotypes may thus contribute to the divergent sepsis outcomes. Temporal dynamics may also be relevant, as early MCP-1-mediated leukocyte infiltration exacerbates hyperinflammation, while sustained MIP1B levels counter subsequent immunosuppression. Further studies delineating cell type-specific mechanisms are warranted to inform therapeutic targeting strategies.

Major strengths of our study include the large sample size, use of multiple MR methods to ensure robustness of findings and leveraging GWAS data to minimize confounding. We performed sensitivity analyses using different statistical models which yielded concordant results, further supporting the reliability of our conclusions. However, we were unable to explore subtype-specific effects as sepsis etiology can differ by infection source, host genetics, and comorbidities (39). Sepsis encompasses a heterogeneous syndrome, and cytokine inflammatory responses likely vary across clinical subtypes (40). Our findings warrant validation in non-European populations as well as mechanistic studies to translate genetic associations into clinical treatments.

Another limitation is that our analysis focused only on circulating cytokine levels, whereas local tissue cytokine milieu may better reflect relevant inflammatory responses in sepsis (41). We were also unable to account for potential dynamic changes in cytokine levels over the clinical course of sepsis. Serial measurement of cytokines could provide further insights into their kinetic profiles during sepsis progression and in response to treatment. Genetic studies also have inherent constraints in inferring causality. Pleiotropy of gene variants can violate MR assumptions, and we applied several sensitivity analyses to minimize this potential bias. Population stratification arising from ancestry differences between cytokine and sepsis GWAS datasets could also confound results. We restricted our analysis to European-only cohorts to mitigate this issue. In addition, there was a challenge in sourcing a dataset that offers sex-stratified or age- stratified information, thereby limiting the ability to verify participants with distinct characteristics.

## Conclusion

In conclusion, our MR study provides evidence for potentially causal relationships of key circulating cytokines with sepsis risk and prognosis. These results highlight modulation of cytokine responses as a promising strategy for sepsis prevention and management. Overall, our findings substantiate the pathogenic role of cytokine dysregulation in sepsis and justify further exploration of immunotherapeutic targets.

## Data availability statement

The original contributions presented in the study are included in the article/**Supplementary Material**. Further inquiries can be directed to the corresponding author.

## Ethics statement

Each study encompassed in the GWASs received approval from the appropriate ethical review committees, and every participant rendered written informed consent. The present investigation exclusively utilized publicly accessible summary-level data; consequently, no further ethical review was mandated for our study.

## Author contributions

FZ: Data curation, Methodology, Supervision, Writing – original draft. JM: Conceptualization, Investigation, Software, Writing – review & editing. DJ: Writing – original draft. JB: Conceptualization, Data curation, Investigation, Methodology, Software, Writing – original draft. QL: Conceptualization, Formal Analysis, Investigation, Project administration, Writing – original draft.
